# Community-based recovery interventions for improving mental health in schizophrenia patients: a scoping review in Southeast Asia

**DOI:** 10.1186/s12888-025-06962-6

**Published:** 2025-05-23

**Authors:** Rohman Hikmat, Suryani Suryani, Iyus Yosep, Rohani Jeharsae, Efri Widianti, Nur Oktavia Hidayati, Titin Sutini, Taty Hernawaty, Aat Sriati, Imas Rafiyah

**Affiliations:** 1 Master of Nursing Program, Faculty of Nursing, Universitas Padjadjaran, Sumedang, Jawa Barat Indonesia; 2 Nursing department, Faculty of Health Science, Universitas ‘Aisyiyah Bandung, Bandung, Indonesia; 3 Department of Mental Health, Faculty of Nursing, Universitas Padjadjaran, Jawa Barat, Sumedang, Indonesia; 4 Faculty of Nursing, Prince of Songkhla University, Pattani Campus, Mueang Pattani District, Pattani, Thailand

**Keywords:** Community, Interventions, Psychosis, Schizophrenia, Recovery

## Abstract

**Background:**

Schizophrenia is a complex mental health condition that affects an individual’s thoughts, perceptions, emotions, and behavior. Recovery from schizophrenia is not solely defined by symptom reduction, but also by the ability to lead a meaningful and fulfilling life within the community. Recovery-oriented approaches emphasize hope, empowerment, social inclusion, and the rebuilding of identity. In Southeast Asia, community-based interventions play a crucial role in supporting individuals diagnosed with schizophrenia through culturally relevant, accessible, and holistic care.

**Aim:**

This scoping review aims to explore the types and characteristics of community-based recovery interventions implemented for individuals diagnosed with schizophrenia in Southeast Asia, specifically in Indonesia, Malaysia, and Thailand.

**Methods:**

A scoping review was conducted following Arksey and O’Malley’s framework. Literature searches were performed in five databases: Scopus, PubMed, CINAHL, Web of Science, and Google Scholar. Keywords included “recovery”, “intervention”, “community-based”, “schizophrenia”, “psychiatric”, and “psychosis”. Inclusion criteria were: (1) studies involving individuals diagnosed with schizophrenia (2), studies conducted in Indonesia, Malaysia, or Thailand (3), articles published in English or Indonesian between 2014 and 2024, and (4) studies focusing on recovery-related outcomes. A qualitative descriptive analysis was used to synthesize findings.

**Results:**

A total of 10 eligible articles were included. The studies explored a variety of community-based recovery interventions, such as psychoeducation programs, empowerment and self-management training, forgiveness therapy, horticultural therapy, web-based cognitive training, and psychosocial interventions including relaxation techniques and mindfulness-based self-awareness. These interventions were found to enhance patients’ knowledge, coping skills, social functioning, and overall quality of life.

**Conclusion:**

Community-based recovery interventions in Southeast Asia show promise in supporting individuals with schizophrenia in their recovery journey. These approaches address not only clinical symptoms but also social and psychological well-being. Further research is needed to evaluate the long-term effectiveness, cultural adaptability, and sustainability of such interventions in diverse community settings.

## Introduction

The diagnosis of Schizophrenia refers to mental health disorders with psychological or behavioral symptoms associated with significant suffering and dysfunction, caused by psychological, social, biological, genetic, physical, or chemical disorders [1]. Schizophrenia is a chronic mental disorder that can cause significant functional disability in various aspects of life. It involves a complex interaction of biological, psychological, social, and environmental factors, and is characterized by a wide range of symptoms including positive symptoms (e.g., hallucinations, delusions), negative symptoms (e.g., affective flattening, avolition), and cognitive impairments [2]. Biological factors include heredity, such as a family history of Schizophrenia increasing a person’s risk of developing schizophrenia [3]. Apart from that, an imbalance of neurotransmitters in the brain, especially dopamine, has been associated with the diagnosis [4]. In the context of schizophrenia, psychological factors such as poor stress tolerance, dysfunctional coping mechanisms, and unresolved early-life trauma have been found to contribute to the onset, exacerbation, and relapse of psychotic episodes. Social factors, including social isolation, unstable interpersonal relationships, and experiences of marginalization, have been associated with schizophrenia, either as potential contributing factors to its development or as consequences of the illness itself [5]. In addition, environmental factors such as habitual use of certain substances, unstable environmental conditions, or chronic stress can also worsen symptoms or increase a person’s risk of experiencing psychosis [6].

In recent years, the concept of recovery in mental health has evolved beyond mere symptom management. Recovery is now understood as a person-centered and holistic process that focuses on empowering individuals to live meaningful lives despite the presence of ongoing mental health symptoms [7]. This includes fostering hope, autonomy, community participation, and the ability to manage one’s condition. Recovery-oriented approaches emphasize the strengths of the individual and support their active involvement in decision-making and goal setting [8]. Mental health recovery can be achieved by minimizing symptoms such as hallucinations and delusions through medical management, while encouraging the belief that individuals with psychosis can lead meaningful lives despite the limitations of their condition [9]. The empowerment approach focuses on the positive attributes and strengths of the individual [10]. This holistic approach to mental health recovery underscores the autonomy of individuals with psychosis in determining and navigating a path to well-being [11].

In Southeast Asia, particularly in Indonesia, Malaysia, and Thailand, challenges persist in accessing appropriate mental health care [12]. Most countries in this region are classified as low- and middle-income, with mental health resources remaining limited [13]. Mental health expenditure is often below 2% of national health budgets, and psychiatric services are predominantly concentrated in institutional settings [14]. Furthermore, a shortage of trained mental health professionals further limits service accessibility and quality. Cultural and religious values such as those derived from Islam in Indonesia and Malaysia, and Buddhism in Thailand also influence the community’s perception of mental illness and the utilization of mental health services [13].

The need to discuss mental health issues in Thailand, Malaysia and Indonesia is very important because there are similarities in various cultural, social and religious aspects between the three. These three countries have significant ethnic diversity, but also have rich and intertwined cultural histories [14]. Value systems inherited from traditional cultures can influence the way individuals and societies interpret, experience, and respond to mental health problems [15]. In addition, religion plays an important role in daily life in these three countries. For example, Buddhism dominates in Thailand, while Islam is the majority religion in Malaysia and Indonesia [16]. These religious principles often influence how individuals and communities deal with stress, trauma, and mental illness, and shape views on mental health care. Furthermore, the social and economic structures between the 3 countries are relatively similar, including strong family patterns and the importance of social relationships in everyday life [17].

Recognizing the burden of mental health conditions and the limitations of institutional care, the World Health Organization has promoted the development of community-based mental health services [18]. These services aim to provide care in less restrictive, more accessible environments, integrated within primary care and supported by trained community health workers [19]. Indonesia, Malaysia and Thailand have developed the concept of community-based health services [13]. Indonesia, Malaysia, and Thailand have initiated such models, focusing on early detection, psychosocial support, and rehabilitation at the community level.

Previous reviews have discussed general aspects of recovery in schizophrenia within Southeast Asia, yet there remains a lack of focused analysis on community-based recovery interventions and their implementation in local contexts [13]. Therefore, this review aims to explore and map the types of community-based recovery interventions implemented in Indonesia, Malaysia, and Thailand. The findings are expected to offer insights into practical approaches and inform mental health professionals and policymakers across similar settings.

## Materials and methods

### Design

This study utilized a scoping review approach based on the methodology proposed by Arksey and O’Malley [20]. This approach involved five main stages, namely: (1) Identification of research objectives (2), Identification of relevant studies (3), Study selection (4), Data extraction, and (5) Mapping, summarizing, and reporting results. The scoping review method was chosen because it allowed researchers to explore the diversity of existing literature on community-based recovery interventions for Schizophrenia patients as a whole in Indonesia, Malaysia, and Thailand. In the context of this research, a scoping review allowed us to identify and describe the various approaches and findings that already existed in the literature, providing a more comprehensive understanding of the topic. The research question was, what were the recovery-based interventions for patients with community-based psychosis problems in Indonesia, Thailand, and Malaysia?

### Search strategy and eligibility criteria

**Fig. 1:**
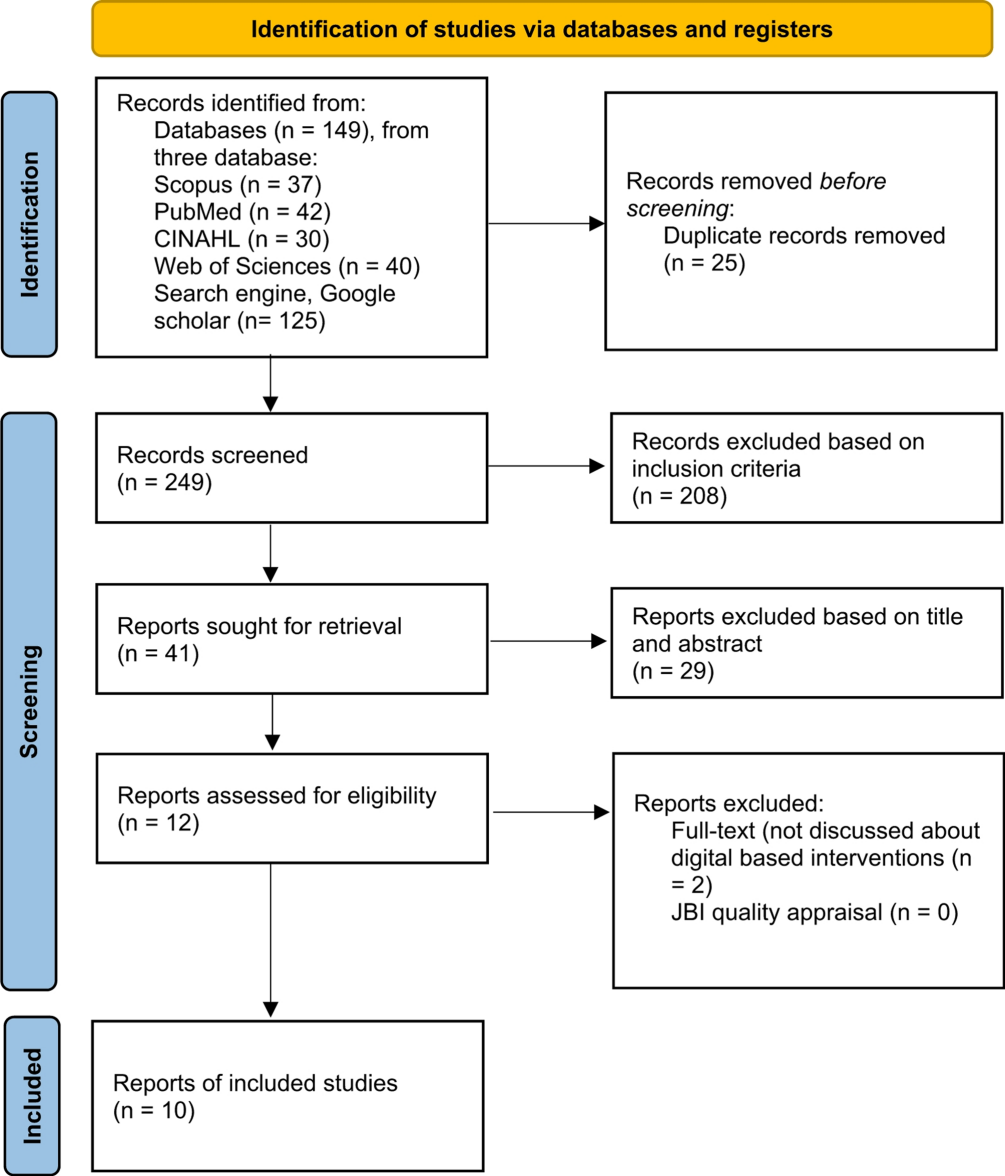
PRISMA flow diagram

A comprehensive literature search was conducted using four major databases: Scopus, PubMed, Web of Science, and CINAHL, as well as the Google Scholar search engine. These databases were selected for their extensive coverage in mental health, medical, and nursing literature. The keywords used in this search included a combination of terms such as *“recovery”*, *“intervention”*, *“community-based”*, and *“schizophrenia”*. Boolean operators (AND/OR) and Medical Subject Headings (MeSH) were applied to improve the accuracy and relevance of the search. The Preferred Reporting Items for Systematic Reviews and Meta-Analyses (PRISMA) Flow Diagram was used to document the article selection process [21] (Fig. 1).

### Inclusion and exclusion criteria

This review adopted the PCC (Population, Concept, Context) framework to guide the inclusion criteria. The Population consisted of individuals diagnosed with schizophrenia or psychosis; the Concept focused on recovery-based interventions; and the Context was community-based settings within Southeast Asia. Studies were included if they met the following criteria: (1) involved patients diagnosed with schizophrenia or other psychotic disorders (2), originated from Indonesia, Malaysia, or Thailand (3), were published between 2014 and 2024 in English or Indonesian, and (4) reported outcomes related to recovery.

Recovery was operationally defined as a person-centered process aimed at improving quality of life, autonomy, social functioning, and empowerment, regardless of the persistence of symptoms. Interventions were classified as recovery-based if they aligned with this definition, as determined through a thematic analysis of each study’s objectives, intervention content, and reported outcomes. These included interventions that promoted empowerment, social inclusion, skill-building, symptom management, or psychological well-being. Studies were excluded if they focused solely on pharmacological treatment without addressing psychosocial recovery goals, did not involve a community-based setting, or lacked sufficient data regarding intervention content and outcomes. Only full-text peer-reviewed articles were included; abstracts-only and gray literature were excluded to maintain quality and rigor.

### Data extraction

Data extraction was carried out using manual tables that included information regarding the authors, study objectives, intervention methods, sample, country, and research results. The manual table aims to describe various research results from the included articles. Data extraction was carried out by two researchers independently and experts in their fields. Wheree differences of opinion occurred between two researchers, these differences were resolved through discussion and joint consensus. However, if after discussion there was still no agreement, a third author was invited to carry out data extraction.

### Quality appraisal

The quality appraisal process was carried out by the authors to evaluate the quality of the study using instruments from the Joanna Briggs Institute (JBI) assessment. The JBI assessment consists of various statements that can be filled in by the authors. JBI instrument for Randomized Controlled Trial (RCT) design, there are 13 statements evaluated. Meanwhile, in the JBI instrument for Quasi-Experiment design there are 9 statements that are evaluated. JBI answer options consist of yes, no, not applicable, and unclear. Yes answers are given a score of 1 and other answers are given a score of 0. Each statement is given a score based on the level of compliance of the study with the specified criteria. A total score is then calculated by adding up the scores for each statement. The quality appraisal process was carried out by two authors independently to ensure accuracy and consistency in the assessment. If there is disagreement, the two authors hold a discussion to determine the score from the JBI assessment. A third authors was invited to provide an assessment if there was still no agreement between the two authors. The minimum score set by the authors as the standard for the articles used is above 75%.

### Data analysis

Data analysis was carried out descriptively qualitatively using a content analysis approach. This analysis aims to describe and summarize the various themes that emerge from the extracted study results. These themes were analyzed in depth to provide a better understanding of community-based recovery interventions for patients with schizophrenia. Data extracted from relevant studies were coded based on themes or concepts that appeared consistently in the literature. After that, the researcher identified main themes from the data by grouping similar or related codes into larger categories. The next stage is analysis and interpretation, where the researcher analyzes the meaning behind the themes and explores their practical implications. Finally, the results of the analysis are compiled in a report that presents the most significant findings and provides an in-depth interpretation of them. Data analysis was carried out by two authors independently and are experts in their fields. During data analysis, differences of opinion were resolved through discussion and deliberation. If there was still no agreement after discussion, a third authors was invited to conduct data analysis.

## Results

Based on the results of initial research conducted by the authors from four databases and one Google Scholar, the authors found 149 articles from 4 databases and 125 articles from the Google Scholar search engine. After eliminating duplicate articles using the Mendeley application, 25 duplicate articles were removed. Following the inclusion criteria, 41 articles that met the inclusion criteria were retained. A further elimination based on the title and abstract resulted in 12 articles that aligned with the research objectives. These 12 articles were read in full, and 10 articles were identified as discussing recovery-based interventions for patients with community-based psychosis problems in Indonesia, Thailand, and Malaysia. These articles were selected based on full-text and assessed for quality using the JBI instrument, with all articles scoring above 75% (Table 1).

**Table 1:** JBI critical appraisal tool

Authors, Published Year	JBI Critical Appraisal Tool	Study Design
[24]	88.9% (8/9)	quasi-experimental
[32]	100% (9/9)	Quasi experiment
[25]	88.9% (8/9)	quasi-experimental
[27]	88.8% (8/9)	Quasi experimental
[28]	84.6% (11/13)	RCT
[30]	88.9% (8/9)	Quasi experimental
[31]	92.3% (12/13)	RCT
[26]	90.9% (10/11)	retrospective cohort study
[33]	88.9% (8/9)	quasi-experimental
[29]	92.3% (12/13)	RCT

Based on the characteristics of the articles, there are 7 articles from Indonesia, 2 articles from Malaysia, and 1 article from Thailand. The samples in these 10 articles ranged from 30 to 104 patients diagnosed with schizophrenia. From the data analysis, several types of recovery-based interventions emerged, namely through empowerment programs, forgiveness therapy, horticulture, psychoeducation, cognitive therapy, and relaxation techniques. These findings are based on the 10 articles and are presented in Table 2.

**Table 2:** Extraction data

No	Authors, Year	Outcomes	Country	Samples	Design	Interventions	Results
1.	[24]	to analyze medication adherence among schizophrenia patients in the Illness Representation Based Education Program (IRBEP)	Indonesia	40 schizophrenia patients	quasi-experimental	the Illness Representation Based Education Program (IRBEP)	The patients in the experimental group had significantly higher medication adherence than those in the control group (t = 6.53, *P* < 0.01). Experiment group had significantly higher medication adherence after attending the IRBEP than that at baseline (t = -6.09, *P* < 0.01).
2.	[32]	to analyze the impact of forgiveness therapy on managing emotions related to violent behavior in individuals with post-restraint schizophrenia.	Indonesia	64 patients with violent behavior schizophrenia	Quasi experiment	forgiveness therapy	This intervention is significant for reducing the risk of violent behavior (*p*-value 0.002)
3.	[25]	to investigate the impact of integrative empowerment intervention on preventing relapse in patients with schizophrenia	Indonesia	70 individuals with schizophrenia	quasi-experimental	Integrative Empowerment Intervention	The intervention had a significant impact on preventing relapse in patients with schizophrenia (*p* < 0.001).
4.	[27]	to analyze the horticultural therapy can effectively improve self-esteem in patients with mental disorders	Indonesia	75 individuals with mental disorders	Quasi experimental	Horticultural Therapy	The results showed a significant difference in the self-esteem scores of patients with mental disorders after intervention (*p*-value of 0.00).
5.	[28]	examines the effects of structured walking participation on QOL, psychosocial functioning and symptoms	Malaysia	104 Chronic patients with schizophrenia	RCT pilots	structured walking participation	There were statistically significant increases in physical functioning (*p* < 0.001), physical role limitations (*p* < 0.05), social functioning (*p* < 0.01), positive (*p* < 0.001) and negative (*p* < 0.01) symptoms, and general psychopathology (*p* < 0.01)
6.	[30]	to analyze the effectiveness of CBR to improve the quality of life of people with schizophrenia.	Indonesia	100 people with schizophrenia 18–56 years old	Quasi experimental	Community-Based Rehabilitation (psychoeducation)	CBR is effective for improving the QoL of people with schizophrenia in the community. CBR is conducted by the health worker and sub-district social welfare worker.
7.	[31]	to determine Web-Based Processing Speed ​​Training and Remediation on global cognition, comparable, and untrained tasks for real world applications.	Malaysia	30 participants of young adults with first episode psychosis aged between 18 and 40 years	RCT	Web-Based Processing Speed ​​Training and Remediation	significant improvement in cognitive functioning and psychosocial including social functioning negative symptoms reduction which is beneficial for recovery in processing speed and untrained skills.
8.	[26]	to identify the effectiveness of Community-Based Mental Health Programs on the recovery of individuals with schizophrenia and psychosis	Indonesia	206 subjects	retrospective cohort study	Community-Based Mental Health Program	Effective on improving recovery in patients
9.	[33]	to examine the effects of mindfulness on hope and recovery among people with schizophrenia	Indonesia	54 patients with schizophrenia	quasi-experimental	Mindfulness therapy	There were significant differences in hope and recovery levels between the two groups with *p*-value < 0.001
10.	[29]	To investigate the efficacy of the Thai Health Improvement Profile intervention for preventing clinically significant weight gain.	Thailand	Fifty-three with early stage psychosis	RCT	Thai Health Improvement Profile intervention	Effective on preventing clinically significant weight gain.

### Empowerment program

The Recovery-Based Empowerment Program (IRBEP) is designed to increase patient understanding and compliance with schizophrenia treatment through seven integrated process components. The program involves a series of sessions, including assessing representation, identifying gaps, creating conditions for conceptual change, introducing substitute information, summarizing, setting goals, and following up via phone [22]. Over time, patients engage in each component, designed to facilitate their recovery with strong community support.

The integrative empowerment intervention module was developed to empower family members of schizophrenia patients. This module was created through a three-stage process, beginning with module development based on a preliminary cross-sectional study identifying factors contributing to the family’s ability to care for the patient [23]. The information from the module is used to create a booklet for family members, and nurses are trained to deliver the module, thereby improving family members’ understanding and skills in caring for those with schizophrenia. This intervention is intended to enhance the effectiveness of family support and facilitate the patient’s recovery.

Additionally, the Community Mental Health Program in Aceh Province involves a team from the Community Health Center, particularly for schizophrenia patients [24]. This program aims to meet the treatment needs of patients at the community level, with a focus on providing mental health services, including diagnosis, treatment, and support for patients and their families. Community involvement is intended to increase accessibility and strengthen social support, aiding patients in their recovery process.

### Horticulture therapy

Horticultural therapy, combined with medication, was implemented over a six-week period. The therapy included four key activities: introducing participants to commonly consumed vegetables, planting and caring for vegetable seeds, harvesting the garden products, and learning to process the harvest [25]. This program aims to provide both psychological and physical benefits, as well as improve participants’ farming skills [26]. A physical training program, involving increased walking durations over three months, also contributed to the overall intervention [27].

Horticultural therapy along with medication use over a six week period. This therapy is designed with four main activities which include the stage of introducing participants to commonly consumed vegetables, then continuing with planting and caring for vegetable seeds [25]. After that, participants will be involved in harvesting the garden products they have planted, and finally, they will learn the stages of processing the harvest. By combining horticultural activities with medication, it is hoped that participants will experience the psychological and physical benefits of this intervention, as well as improve their skills in farming. In addition, there is a physical training program carried out by increasing the duration of walking exercise for three months, starting from 20 min per session up to 40 min per session [26].

The Thai Health Improvement Profile (T-HIP) intervention encompasses a comprehensive training program designed to equip healthcare professionals with the necessary tools and strategies for effective health improvement interventions. The training includes the utilization of the Health Improvement Profile tool, which recommends interventions for each rephrased item of the Thai Health Improvement Profile. Strategies are outlined for engaging patients in making effective changes in health-related behaviors according to the transtheoretical model of change, motivational interviewing approaches, formulation of individualized care plans, and follow-up actions. The theoretical aspects of the training and a review of the T-HIP tool/handbook are delivered over a 3-hour session. In the afternoon, nurses spend 1.5 h practicing using the T-HIP tool to conduct comprehensive assessments and an additional hour practicing collaborative care plan development [27].

### Psychoeducation

The psychoeducation program is delivered to patients and their caregivers in six different sessions, carried out within a certain predetermined time span [28]. The timing of these sessions may vary depending on the policies and schedule determined by the program organizer. In general, each session usually lasts one to two hours, depending on the complexity of the material presented and the interaction between participants and the material presenter. Each session is conducted by primary care physicians and community mental health nurses, with the aim of providing comprehensive information about various aspects of schizophrenia. According to the program, participants will attend these sessions sequentially, with the hope that they will gain a better understanding of the condition of schizophrenia and how to manage it effectively within the specified time.

### Cognitive therapy

Web-based cognitive training to help patients overcome the cognitive impairments often associated with schizophrenia [29]. This training is conducted using commercial software proven to be effective in cognitive remediation. This training is usually carried out regularly over a period of time, which can range from several weeks to several months, depending on the treatment plan drawn up for each patient. Patients undertake online cognitive training sessions with support from trained mental health professionals, with the hope that they will experience improvements in their cognitive function and overall speed up their recovery from schizophrenia.

### Forgiveness therapy

This intervention is forgiveness therapy aimed at patients who have had symptoms of schizophrenia for less than one year, and have committed violent behavior in less than 3 weeks. These patients have a regular habit of taking medication and schedule regular check-ups with health services [30]. This therapy aims to help patients overcome negative emotions and behaviors that trigger violence. In contrast to conventional Cognitive Behavioral Therapy (CBT) theory, this therapy proposes that change will not occur through emotional habituation of difficult stimuli, but rather by restructuring and transforming difficult emotional processing processes. This forgiveness therapy involves four phases, namely the phase of expressing anger, the phase of deciding to forgive, the phase of forgiveness, and the phase of discovery and liberation from emotional prison.

### Relaxation therapy

Interventions involve various techniques such as relaxation techniques, self-awareness, self-compassion, and acceptance of the patient’s condition. These intervention sessions are held regularly, namely once a week, with the main aim of reducing negative self-assessment and improving the patient’s quality of life. Through these exercises, it is hoped that patients can gain skills to manage stress, increase awareness of their condition, and develop a better attitude towards themselves, all of which contribute to their recovery process from schizophrenia disorders.

Mindfulness therapy has emerged as a promising approach in the recovery journey of patients with schizophrenia [31]. It involves cultivating present-moment awareness and acceptance of one’s thoughts, feelings, and bodily sensations without judgment. Incorporating mindfulness techniques such as meditation, breathing exercises, and body scans, this therapy aims to enhance individuals’ ability to cope with distressing symptoms and improve overall well-being. Research suggests that mindfulness-based interventions can be beneficial for individuals with schizophrenia by reducing symptoms such as hallucinations, delusions, and emotional dysregulation.

## Discussion

The results of this scoping review show that there are 10 articles that discuss recovery-based interventions with community approaches for recovery in schizophrenia patients. All articles indicate that recovery-based interventions can effectively facilitate the recovery process in schizophrenia patients. This approach emphasizes patient empowerment, giving individuals greater control over their own recovery journey, thereby increasing motivation and independence [32]. Intervention activities, such as psychoeducation, horticultural therapy, cognitive training, and others, are designed to address various aspects related to schizophrenic disorders, ranging from symptom management to social and environmental factors. Additionally, these interventions often involve family and community, creating a supportive environment that is essential in the recovery process. Compared to traditional approaches focused primarily on symptomatic treatment, recovery-based interventions lead to better outcomes in improving the quality of life, independence, and psychosocial well-being of schizophrenia patients [33].

### Empowerment-based interventions

Empowerment-based interventions have proven effective in increasing patient understanding and adherence to schizophrenia treatment. These interventions focus on patients’ holistic needs, involving the patient, their family, and strong community support [34]. This is consistent with recovery theory in mental health, which emphasizes the importance of collaboration between patients, families, and communities in the recovery process. The intervention’s seven components—such as assessment, identification of gaps, and follow-up on goals provide a comprehensive framework to facilitate patient recovery. Empowerment-based interventions require integrated modules, providing clear guidance for nurses and family members in caring for schizophrenia patients [23]. Approaches that engage the patient, family, and community tend to be more effective in improving recovery outcomes for schizophrenia patients than those focusing solely on medical treatment [35].

### Horticultural therapy

Horticultural therapy, when combined with medication, shows potential to be an effective intervention in helping patients recover. Over a six-week period, components such as introducing, planting, harvesting, and processing garden products offer patients the opportunity to engage in activities that are both physically and psychologically beneficial [36]. This active involvement increases participants’ sense of accomplishment, self-confidence, and practical farming skills [37]. The integration of horticultural therapy with medication creates a holistic approach that addresses both physical and mental well-being [38]. The interaction between physical activity, nature, and medication provides comprehensive support in the recovery process [39]. Moreover, a physical exercise program, gradually increasing the duration of walking, contributes positively to the physical health of participants [40]. These findings align with previous research demonstrating the benefits of horticultural therapy and physical activity in enhancing quality of life and mental well-being [41].

### Psychoeducation

A psychoeducational program delivered to patients and their caregivers in six sessions demonstrated the potential to be effective in increasing understanding of schizophrenia and its management. This intervention provides an opportunity for participants to obtain comprehensive information about various aspects of schizophrenia, including definition, symptoms, management, recovery, family roles, and stress management [42]. These interventions can promote a better understanding of their condition and develop the skills necessary to manage it effectively [43]. The administration of sessions by primary care physicians and community mental health nurses provides additional advantages in terms of extensive experience and understanding of the schizophrenic condition [44]. These results are in line with previous research showing that psychoeducation is effective in increasing understanding of mental conditions and increasing patient engagement in their care [45].

### Web-based cognitive training

Web-based cognitive training offers an innovative approach to addressing cognitive impairments associated with schizophrenia. This intervention allows patients to engage in cognitive remediation at home, enhancing accessibility and patient involvement [46]. Commercial software, which has shown effectiveness in cognitive training, provides a robust empirical basis for the intervention [47]. Previous research supports the efficacy of cognitive training in improving cognitive function in schizophrenia patients, thus accelerating recovery [48, 49]. The flexibility of this approach enables patients to engage with the program independently, with guidance from trained professionals, facilitating a more personalized experience. These findings are consistent with research demonstrating that cognitive training significantly improves cognitive function in schizophrenia patients [50].

### Relaxation techniques

Relaxation techniques, combined with self-awareness, self-compassion, and acceptance, have proven effective in supporting the recovery of schizophrenia patients. Techniques such as relaxation and self-awareness have long been known to assist individuals in managing stress and improving their mental well-being [51]. This intervention provides space for self-reflection, allowing patients to develop a positive attitude towards themselves and reduce negative self-judgment, which is crucial for recovery [52, 53]. By offering a holistic approach to overcoming schizophrenia symptoms, this intervention improves overall quality of life [54]. These findings support previous research showing that combining relaxation and self-awareness techniques can significantly enhance mental well-being and quality of life for schizophrenia patients [24].

### Cultural and socioeconomic context

While the recovery-based interventions discussed in this review have shown positive outcomes, it is important to consider the socioeconomic and cultural contexts in which they are implemented. Many of the interventions, such as psychoeducation, cognitive training, and horticultural therapy, have roots in Western practices and may not always align perfectly with the cultural norms and values of Southeast Asian countries. There is a growing recognition that healthcare programs imported from Western countries must be adapted to local cultural contexts to be truly effective [24]. For instance, in countries like Indonesia, Malaysia, and Thailand, family plays a central role in the caregiving process, and interventions that involve family members are often more effective than those that do not. Empowerment-based approaches, which integrate family and community, may be more culturally appropriate in these settings, where collective support is often emphasized over individual autonomy [55].

In Southeast Asia, where there are diverse cultural perspectives on mental health, it is crucial to ensure that interventions resonate with local beliefs and practices. For example, community-based approaches that focus on social harmony and collective well-being might be more successful than Westernized models that emphasize individual empowerment. Tailoring interventions to fit the cultural understanding of schizophrenia can increase their acceptability and effectiveness [56]. Additionally, the socioeconomic factors, such as access to healthcare, education, and technology, can influence the success of interventions like web-based cognitive training. In regions where access to technology is limited, these programs may require adjustments or alternative delivery methods to ensure accessibility for all patients [57]. Therefore, while Western-derived recovery programs have proven beneficial, their integration into Southeast Asian contexts requires careful consideration of local cultural values, socioeconomic factors, and healthcare systems. Adapting these interventions to suit local needs can enhance their effectiveness and ensure better outcomes for schizophrenia patients in these regions.

#### Limitations

A limitation in this study is that the publication period is limited to the last 10 years (2014–2024), this means that this study cannot discuss the results of previous research which discussed recovery based interventions. Then, another limitation is that the database and search engines used are limited. This research focuses on research in three countries, namely Indonesia, Malaysia and Thailand. The national databases in these three countries need to be further expanded to increase the trustworthiness of the study.

## Conclusion

Based on the results of this scoping review, the authors found ten articles that investigated various interventions in the management of schizophrenia. Interventions involving a combination of psychosocial, psychological, and physical approaches demonstrate effectiveness in improving patients’ understanding, skills, and quality of life. The types of interventions found included empowerment programs, forgiveness therapy, psychoeducation programs, horticultural therapy, physical exercise, web-based cognitive training, as well as psychosocial interventions such as relaxation techniques and self-awareness. The importance of integrating various interventions to treat schizophrenia symptoms and providing holistic support to patients and their families is also emphasized.

The nursing implication of these results is the need for nurses to support the implementation of these interventions, including providing education, accompanying patients, and involving families in care. Recommendations for further research include exploring the effectiveness of each type of intervention, as well as conducting more in-depth research regarding its effect on the quality of life and recovery process of schizophrenia patients through a randomized control trial design.

## Data Availability

All data generated or analyzed during this study are included in this published article.
